# Caloric restriction in C57BL/6J mice mimics therapeutic fasting in humans

**DOI:** 10.1186/1476-511X-5-13

**Published:** 2006-05-18

**Authors:** Lisa B Mahoney, Christine A Denny, Thomas N Seyfried

**Affiliations:** 1 Biology Department, Boston College, Chestnut Hill, MA, USA

## Abstract

**Background:**

Caloric restriction (CR) has long been recognized as a dietary therapy that improves health and increases longevity. Little is known about the persistent effects of CR on plasma biomarkers (glucose, ketone bodies, and lipids) following re-feeding in mice. It is also unclear how these biomarker changes in calorically restricted mice relate to those observed previously in calorically restricted humans.

**Results:**

Three groups of individually housed adult female C57BL/6J (B6) mice (n = 4/group) were fed a standard rodent chow diet either: (1) unrestricted (UR); (2) restricted for three weeks to reduce body weight by approximately 15–20% (R); or (3) restricted for three weeks and then re-fed unrestricted (*ad libitum*) for an additional three weeks (R-RF). Body weight and food intake were measured throughout the study, while plasma lipids and levels of glucose and ketone bodies (β-hydroxybutyrate) were measured at the termination of the study. Plasma glucose, phosphatidylcholine, cholesterol, and triglycerides were significantly lower in the R mice than in the UR mice. In contrast, plasma fatty acids and β-hydroxybutyrate were significantly higher in the R mice than in the UR mice. CR had no effect on plasma phosphatidylinositol levels. While body weight and plasma lipids of the R-RF mice returned to unrestricted levels upon re-feeding, food intake and glucose levels remained significantly lower than those prior to the initiation of CR.

**Conclusion:**

CR establishes a new homeostatic state in B6 mice that persists for at least three weeks following *ad libitum *re-feeding. Moreover, the plasma biomarker changes observed in B6 mice during CR mimic those reported in humans on very low calorie diets or during therapeutic fasting.

## Background

Caloric restriction (CR) has long been recognized as a natural therapy that improves health and extends longevity in humans and rodents [1-7]. CR diminishes inflammation and oxidative stress that occurs from aging by decreasing the production of reactive oxygen species [1,8,9]. In rodents and primates, CR lowers plasma insulin, cholesterol, triglycerides, and insulin-like growth factor (IGF-1) levels, while elevating plasma high-density lipoprotein (HDL) levels [10-14]. These changes in plasma metabolites reduce risk for atherosclerosis, diabetes, and obesity [15]. Additional health benefits of CR likely result from reduced glucose levels and elevated ketone bodies (β-hydroxybutyrate), which reduce oxygen free radicals and increase the ΔG' of ATP hydrolysis [6,14,16,17].

Numerous studies in humans have used fasting as a treatment for obesity, diabetes, and cancer [18-21]. Therapeutic fasting differs from starvation in mobilizing fat rather than protein for energy. Very low calorie diets (approximately 300 kilocalories per day) often produce effects that are similar to those seen during therapeutic fasting [22-24]. During the initial stages of a full food fast (water only), blood glucose levels are initially maintained by the mobilization of stored glycogen (glyogenolysis). As glycogen stores become depleted, the body gradually transitions to fatty acids and ketone bodies for additional energy. Although gluconeogenesis also increases, this is insufficient alone to provide enough energy, especially for the brain [21,25-28]. Continued fasting decreases total plasma cholesterol, low-density lipoprotein (LDL) levels, and triglycerides, while elevating fatty acids [20,29,30]. Since the brain does not generally metabolize fatty acids for energy [31], ketone bodies provide the largest source of energy for the brain during prolonged fasting [32]. Ketone bodies are a more efficient energy source than either glucose or fatty acids because they are more reduced (a greater hydrogen/carbon ratio) than pyruvate and do not uncouple the mitochondrial proton gradient as occurs with fatty acid metabolism [17].

Few studies have examined the longer-term effects of CR or fasting on the concentration of plasma metabolites following *ad libitum *re-feeding. Most previous studies examined biomarker changes following brief periods of re-feeding (approximately 4 days) [33-35]. In general, re-feeding restored levels of cholesterol, triglycerides, glucose, ketone bodies, fatty acids, and body weight to the levels seen prior to the initiation of CR or fasting [15,18,34-37]. No prior studies, to our knowledge, have determined to what extent CR-induced plasma biomarker changes persist in mice following *ad libitum *re-feeding for several weeks. It is also unclear how plasma biomarker changes in mice under CR relate to those observed in humans under food restricted diets.

In this study, we found that three weeks of moderate CR in adult female C57BL/6J (B6) mice significantly reduced plasma glucose, cholesterol, triglycerides and body weight, while elevating fatty acids and ketone bodies. Although *ad libitum *re-feeding for three weeks restored body weight and most CR-induced biomarker changes, food intake and glucose levels remained lower in the R-RF mice than in the UR mice. These findings suggest that the health benefits of CR persist for at least three weeks in B6 mice thus producing a physiological state more energy efficient than that prior to CR. Moreover, the plasma biomarker changes found in B6 mice during three weeks of CR mimic those reported in humans during a very low calorie diet or therapeutic fasting.

## Results

Compared to the UR mice, the R mice were healthier and more active as assessed by ambulatory and grooming behavior. There were no signs of vitamin or mineral deficiency in the R mice according to standard criteria [38]. These findings are consistent with the well-established health benefits of mild to moderate CR in rodents and why it is unnecessary to supplement with vitamins and minerals during short-term (up to 12 weeks) CR studies [3,4,39-41].

### Influence of caloric restriction and re-feeding on food intake and body weight

**Figure 1:**
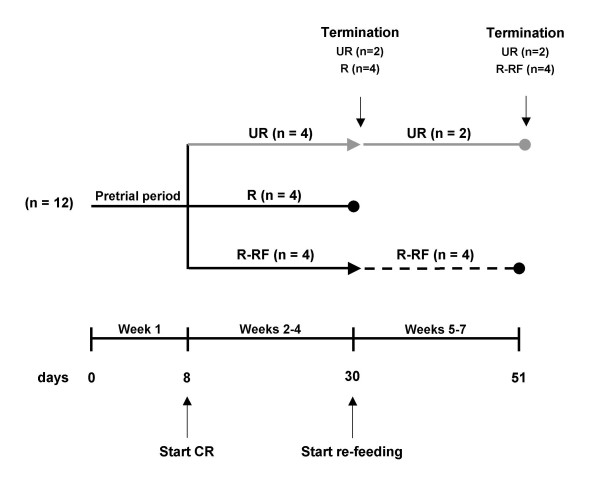
Flow chart of the study design. Body weight and food intake were measured every other day over the seven day pre-trial period. All mice received food *ad libitum *during the pre-trial period. After the pre-trial period, the mice were divided into three groups (n = 4 mice/group) where the average body weight of each group was similar. The mice in each group were then fed the same diet in different amounts: 1) the standard chow diet unrestricted (UR), 2) the standard chow diet restricted to achieve an approximate 15–20% body weight reduction from the pre-trial weight (R), or 3) the standard chow diet restricted to achieve an approximate 15–20% body weight reduction from the pre-trial weight for a period of three weeks, followed by unrestricted re-feeding for a period of three weeks (R-RF). Each mouse in the R and the R-RF groups served as its own control for body weight reduction as previously described [16]. Based on food intake and body weight during the pre-trial period, food in the R and the R-RF groups was reduced until each mouse achieved the target weight reduction of approximately 15–20%. The study was terminated and plasma was collected for two UR mice and four R mice on day 30, and for the remainder of the mice on day 51.

**Figure 2:**
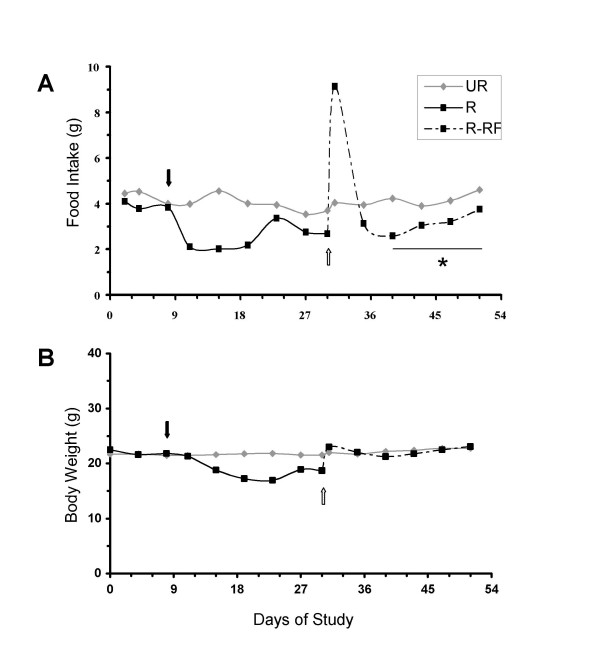
Influence of CR and re-feeding on food intake (A) and body weight (B). Values are expressed as means and 4–8 mice were analyzed in each group. The black arrow indicates the initiation of CR on day 8. The white arrow indicates the initiation of *ad libitum *re-feeding on day 30. The * indicates that the food intake average of the days 38 to 50 of re-feeding for the R-RF mice was significantly less than their food intake prior to initiation of CR, as determined by the paired *t*-test.

Adult virgin female mice were used for this study because their food intake and body weights are relatively stable from about 120 to 170 days of age (Figs. 1, 2A and 2B). The average total food intake for the UR group during weeks 2–4 was 86.9 ± 2.2 g (n = 4), and over the next three weeks was 87.5 g (n = 2). The amount of food provided for the R mice was initially 60% (40% restriction) of that eaten prior to the initiation of CR (pretrial period). The amount of food given to the R mice was then adjusted each day (± 5%) to achieve a final body weight reduction of approximately 15%. The average total food intake for all restricted mice (n = 8) during the three week restriction period was 52.2 ± 1.5 g. This represents an overall average total food restriction of approximately 40% over the three week period. Body weight was chosen as an endpoint for CR rather than food intake because body weight is a more stable variable than food intake, which differs significantly among mice, even within the same strain. [4,16]. A 15% body weight reduction achieved by a 40% restriction in food represents a moderate caloric restriction for adult mice [4]. Body weight was reduced in the R group at day 15 and remained significantly lower than that of the UR group (p < 0.01) until day 30. On the day of re-feeding, the R-RF mice binge ate and consumed approximately twice as much food (8.5 g/day/mouse) as they did during the pretrial period (about 4.2 g/day/mouse). Food intake in the R-RF mice decreased rapidly, but remained greater than that of the UR mice until day 33 (Fig. 2A). The average total food intake for the R-RF group (n = 4) for the three week re-feeding period, including the three day binge period, was 76.9 ± 2.0 g. Interestingly, the food intake following the binge period (from day 38 through the end of the study) was less in the R-RF mice (3.35 g/day/mouse (n = 4)) than in the UR mice (4.17 g/day/mouse (n = 2) (Fig. 2A)). The R-RF mice also ate significantly less food per day during this period than they did during the pretrial period (4.08 g/day/mouse (n = 4) (p < 0.05)) as determined by the paired *t*-test. Despite reduced food intake, the body weights of the R-RF mice returned to the levels observed during the pre-trial period and were similar to the body weights of the UR mice (Fig. 2B).

### Influence of CR and re-feeding on plasma glucose and β-hydroxybutyrate levels

Glucose levels were 41% less in the R mice than in the UR mice (Table 1). Although the glucose levels increased following re-feeding in the R-RF mice, the levels remained significantly lower than those in the UR mice. Plasma β-hydroxybutyrate levels were 367% greater in the R mice than in the UR mice (Table 1). Once re-fed, the plasma β-hydroxybutyrate levels for the R-RF mice returned to those measured in the UR mice. These findings are consistent with our previous studies that β-hydroxybutyrate levels are increased under CR and that circulating β-hydroxybutyrate levels are inversely related to circulating glucose levels [4,7,16].

### Influence of CR and re-feeding on neutral and acidic lipids

**Figure 3:**
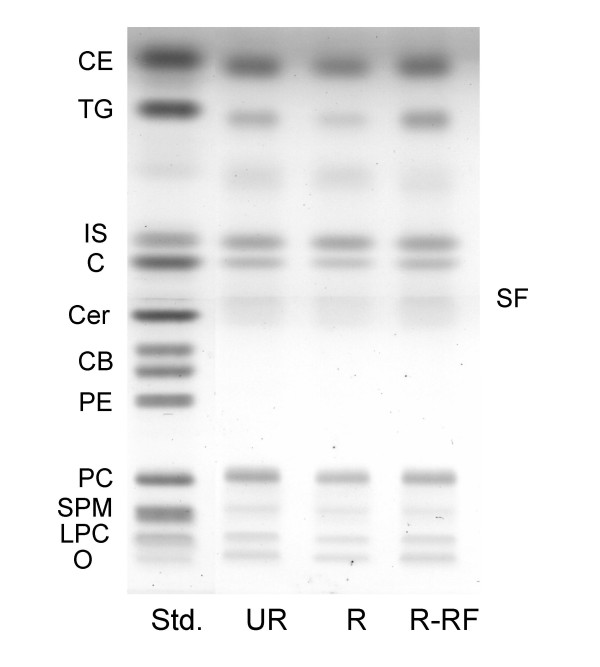
HPTLC of plasma neutral lipids in B6 mice. The amount of neutral lipids spotted per lane was equivalent to 2.5 μl of plasma. The plate was developed as described in the Methods. CE, cholesterol esters; TG, triglycerides; IS, internal standard; C, cholesterol; Cer, ceramide; CB, cerebrosides (doublet); PE, phosphatidylethanolamine; PC, phosphatidylcholine; SM, sphingomyelin; LPC, lysophosphatidylcholine; O, origin; and SF, solvent front of the first developing solvent system.

**Figure 4:**
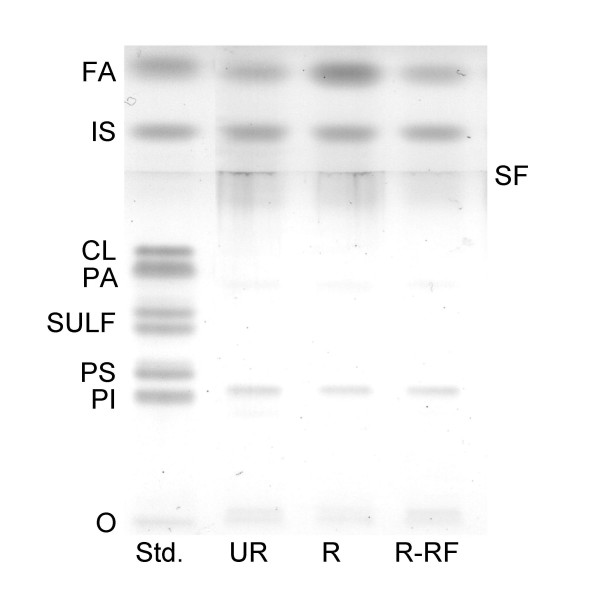
HPTLC of plasma acidic lipids in B6 mice. The amount of acidic lipids spotted per lane was equivalent to 15 μl of plasma. The plate was developed as described in the Methods. FA, fatty acids; IS, internal standard; CL, cardiolipin; PA, phosphatidic acid; Sulf, sulfatides (doublet); PS, phosphatidylserine; PI, phosphatidylinositol; O, origin; and SF, solvent front of the first developing solvent system.

The influence of CR and re-feeding on the qualitative and quantitative distribution of plasma neutral lipids and acidic lipids in B6 mice is shown in Figs. 3 and 4, respectively, and in Table 1. Triglycerides, cholesterol, and phosphatidylcholine were significantly reduced, while fatty acids were significantly elevated in the R mice when compared to the UR mice. All plasma lipids in the R-RF mice returned to the levels seen in the UR mice. In contrast to phosphatidylcholine, which was reduced in the R mice and returned to normal levels in the R-RF mice, CR and re-feeding had no effect on plasma levels of phosphatidylinositol. Although sphingomyelin and lysophosphatidylcholine were detected in the plasma of all groups (Fig. 3), no statistically significant differences were found among the groups for these lipids due to sample variability. It is important to mention that the solvent front (SF) does not include lipids, but contains slight impurities from the organic solvents used in the developing system.

**Table 1:** Influence of Caloric Restriction and Re-feeding on Plasma Metabolites in B6 Mice^a^

Metabolites	UR	R	Difference(%)	R-RF	F^d^(2,9)
Glucose^b^	15.5 ± 0.86	9.1 ± 1.83**	41	12.5 ± 1.61*	18.3
					
β-hydroxybutyrate^b^	0.3 ± 0.12	1.4 ± 0.13**	367	0.3 ± 0.05	141.4
					
*Neutral lipids *^c^					
Triglycerides	2.4 ± 0.63	1.0 ± 0.16*	-58	2.5 ± 1.00	6.1
Cholesterol	0.5 ± 0.23	0.2 ± 0.06*	-60	0.4 ± 0.14	3.7
Phosphatidylcholine	2.8 ± 0.53	1.7 ± 0.25**	-39	2.8 ± 0.30	10.3
					
*Acidic Lipids *^c^					
Fatty acids	0.3 ± 0.08	0.8 ± 0.05**	167	0.3 ± 0.05	69.7
Phosphatidylinositol	0.2 ± 0.03	0.2 ± 0.01	0	0.2 ± 0.03	1.2

^a ^Values represent the mean ± 95% CI. Four independent samples were analyzed per group.

^b ^Values are expressed as mM.

^c ^Determined from densitometric scanning of HPTLC as shown in Figures 3 and 4. Values are expressed as mg lipid/ml plasma.

^d^ /F/ ratio and degree of freedom (df) obtained from one way analysis of variance.

Astericks indicate that the value is significantly different from that of the control at the * p < 0.05 and ** p < 0.01 as determined by ANOVA followed by Fisher's PLSD

## Discussion

Reliable biomarkers can be useful for gauging the degree and efficacy of CR as a therapy for a variety of diseases to include: aging, neurological and neurodegenerative diseases, and cancer. Our data show that reductions in plasma glucose, cholesterol, phosphatidylcholine, and triglycerides, combined with elevations of ketone bodies (β-hydroxybutyrate), and fatty acids, are robust biomarker changes for CR in the B6 mouse. Similar changes in glucose and ketone bodies have been observed in other mouse strains and rodent models under CR [6,7,16,42]. Cholesterol esters, sphingomyelin, and lysophosphatidylcholine are less reliable plasma biomarkers of CR due to variability between individual mice. It is interesting to note that phosphatidylinositol levels were unchanged as a result of CR and re-feeding, suggesting that this lipid might serve as an internal control for assessing the degree of change in other plasma biomarkers of CR.

Little is known about the persistent effects of CR on plasma biomarkers (glucose, ketone bodies, and lipids) following re-feeding in mice. Previous studies showed that CR-induced biomarker changes return to levels seen prior to CR following brief periods of *ad libitum *re-feeding (approximately 4 days) [34,35]. Our results showed that all biomarkers in the R-RF mice returned to the levels seen prior to CR with the exception of food intake and glucose levels. Since blood glucose levels are directly related to food intake, the persistent reduction in blood glucose reflects the reduction in food intake. These findings suggest that the R-RF mice have established a new, more efficient homeostatic state, in which reduced food intake can maintain body weight similar to that seen during the pretrial period.

Our results are in agreement with those of other investigators [43-45] who observed an energy conservation mechanism due to a decrease in thermogenesis, allowing less energy to be lost as heat and more accumulated as protein, fat, and glycogen. This increase in metabolic efficiency could be the result of several factors involved in homeostasis, but most likely is the result of a decrease in total heat production of the thermoregulatory system. In addition to increasing ATP production, while reducing oxygen consumption, ketone body metabolism also reduces production of damaging free radicals [17,46]. For these and other reasons, Veech has described ketone bodies as "super fuel" [17]. We suggest that the health benefits of CR result in part from a bioenergetic mechanism made more efficient through an increase in ketone body metabolism coupled with a decrease in glucose metabolism. Further studies will be needed to identify those physiological changes within the mitochondria that contribute to or underlie the more efficient metabolic state.

The physiological relationship between CR in mice and humans is unclear. Although rodents and other animals can be maintained on calorie-restricted diets for prolonged periods [47], this draconian dietary practice is impractical in humans. Since the basal metabolic rate of mice is about seven times that of humans [48], it is unlikely that similar degrees of CR will have similar physiological effects in man and mouse. Indeed, a review of the literature generally shows that the plasma biomarker changes we observed in B6 mice, which received approximately 60% of the food given to the UR mice on a daily basis, are generally similar to those observed previously in humans during very low calorie diets or during "water only" therapeutic fasting (Table 2). While prolonged therapeutic fasting (for one to three weeks) can be healthy for some humans [49], severe food deprivation beyond a few days is unhealthy in rodents due to increased oxidative stress [50]. Our findings indicate that moderate CR in B6 mice mimics very low calorie diets or therapeutic fasting in humans. Hence, the numerous health benefits documented in mice following CR may be experienced in humans on very low calorie diets or during periodic therapeutic fasting.

**Table 2:** Influence of Fasting/Very Low Calorie Diet on Plasma Metabolites in Humans^a^

Metabolites	Length (days)	Unrestricted	Fasted	Difference (%)	References	
Glucose	21	7.06	4.39	-38	Owen et al. 1998	[19]
	21–35	5.11	3.89	-24	Streja et al. 1977	[58]
						
β-hydroxybutyrate	2	0.03	1.67	5.E+03	Pan et al. 2000	[59]
	3	0.03	3.15	1.E+04		
	21	0.19	4.60	2.E+03	Owen et al. 1998	[19]
	21–35	0.11	4.56	4.E+03	Streja et al. 1977	[58]
						
*Neutral lipids*						
Triglycerides	7	3.46	2.50	-28	Balazsi et al. 1983	[29]
	14	3.46	1.77	-49		
	28	1.13	0.95	-16	Shoji et al. 1992	[30]
						
Cholesterol	7	4.90	6.73	37	Savendahl and Underwood 1999	[20]
	7	5.48	5.16	-6	Balazsi et al. 1983	[29]
	14	5.48	4.36	-20		
	14	5.14	4.01	-22	Schouten et al. 1981	[60]
	15	5.39	4.43	-18	Cominacini et al. 1991	[61]
	28	5.22	4.21	-19	Shoji et al. 1992	[30]
						
LDL Cholesterol	7	2.91	2.96	2	Balazsi et al. 1983	[29]
	14	2.91	2.43	-16		
						
Phosphatidylcholine	7	2.21	2.39	8	Savendahl et al. 1997	[62]
						
*Acidic Lipids*						
Fatty acids	21	0.84	1.19	42	Owen et al. 1998	[19]
	21–35	0.51	0.85	67	Streja et al. 1977	[58]

^a ^Values are expressed as mM.

## Conclusion

CR establishes a new homeostatic state in B6 mice that persists for at least three weeks following *ad libitum *re-feeding. Moreover, the plasma biomarker changes observed in B6 mice during CR mimic those reported in humans on very low calorie diets or during therapeutic fasting.

## Methods

### Mice

C57BL/6J (B6) mice were obtained from the Jackson Laboratory (Bar Harbor, ME, USA) and were propagated in the Boston College Animal Care Facility. Adult female mice were used and were housed individually in plastic cages with filter tops containing Sani-Chip bedding (P.J. Murphy Forest Products Corp., Montville, NJ, USA. Cotton nesting pads were provided to all mice for warmth for the duration of the experiment, and room was maintained at 22°C on a 12 h light – 12 h dark cycle. The procedures for animal use were in strict accordance with the NIH Guide for the Care and Use of Laboratory Animals and were approved by the Institutional Animal Care Committee.

### Caloric restriction, body weight and food intake measurements

All mice received PROLAB RMH 3000 chow (LabDiet, Richmond, IN, USA). This contained a balance of mouse nutritional ingredients and delivers 4.4 kcal g^-1 ^gross energy, where fat, carbohydrate, protein, and fiber comprised 55, 520, 225, and 45 g kg^-1 ^of the diet, respectively. A total of 12 singly caged, adult female B6 mice were used for the study. The mice were matched for age (120 ± 8 days), sex (virgin females), and body weight (22.0 ± 1.0 g). The experimental design for implementation of CR and re-feeding is outlined in Fig. 1. Body weight and food intake measurements were taken at approximately the same time of day (11:00 AM – 1:00 PM) for all mice. Body weight was measured every two days for the UR and R mice. The R-RF mice were weighed daily during the binge period, and every two days thereafter. Food intake for the UR mice was determined daily by subtracting the weight of the food pellets remaining in the food hopper after two days from the initial amount given (approximately 80 g) and dividing the difference by two. For mice in the R and R-RF groups, weighed food pellets were dropped directly into each cage for easy access. Water was provided *ad libitum *for all mice.

### Glucose and β-hydroxybutyrate measurements

Mice were sacrificed with isofluorane (Halocarbon Laboratories, River Edge, NJ, USA) and blood was collected into heparinized tubes from either the retro-orbital sinus or the heart. The blood was centrifuged at 6,000 × *g *for 10 min, the plasma was collected, and aliquots were stored at -80°C until analysis. Plasma glucose concentration was measured spectrophotometrically using the Trinder Assay (Sigma-Aldrich, St. Louis, MO, USA). The ketone body β-hydroxybutyrate was measured enzymatically using the Stanbio β-Hydroxybutyrate LiquiColor^® ^assay kit (Stanbio, Boerne, TX, USA).

### Lipid isolation and purification

Acidic and neutral lipids were isolated and purified from plasma using modifications of previously described procedures [51-53]. Briefly, total lipids were extracted by adding chloroform (CHCl_3_) and methanol (CH_3_OH) to an aliquot of plasma to produce a ratio of CHCl_3 _: CH_3_OH : aqueous plasma (30:60:8 by vol). The plasma volume was used to calculate the volume of CHCl_3 _and CH_3_OH needed to achieve the ratio. Solvent A (CHCl_3 _: CH_3_OH : dH_2_0; 30:60:8 by vol) was added to increase the total volume of each sample. The solution was placed on a magnetic stirrer at room temperature overnight and then centrifuged for 20 min at 1200 × *g*. The supernatant was collected and the pellet was washed with solvent A, placed on the stirrer for 30 min, and centrifuged as before. The supernatants were combined.

The neutral lipids and acidic lipids were purified using DEAE-Sephadex (A-25, Pharmacia Biotech, Upsala, Sweden) column chromatography as previously described [52,53]. The total lipid mixture was applied to a DEAE-Sephadex column with a bed volume of 1.2 ml that had been equilibrated prior with solvent A. Neutral lipids were eluted from the column by washing two times with 20 ml of solvent A. Acidic lipids were then eluted from the column with 30 ml of solvent B (CHCl_3 _: CH_3_OH : 0.8 M Na acetate, 30:60:8 by vol). The neutral lipid fraction was dried using rotary evaporation, washed with 1 ml dH_2_0 and 4 ml CHCl_3_:CH_3_OH (2:1 by vol), and centrifuged at 1200 × *g *to partition neutral lipids into the Folch lower phase [54,55]. The upper phase was removed and the lower phase was washed once with the Folch pure solvent upper phase [PSUP] (CHCl_3_:CH_3_OH:dH_2_0, 3:48:47 by vol) and centrifuged again at 1200 × g for 15 min. The upper phase was removed and the lower phase was then evaporated under a stream of nitrogen, re-suspended in 5 ml of CHCl_3_:CH_3_OH (2:1 by vol), and stored at 4°C.

The acidic lipid fraction was evaporated under vacuum and 7 ml of CHCl_3_:CH_3_OH (1:1 by vol) was added. CHCl_3 _(3.5 ml) and dH_2_0 (2.6 ml) were added, and the mixture was inverted, vortexed, and centrifuged to partition acidic lipids into the lower phase. The upper phase was removed and the lower organic phase was washed once with 4.5 ml of the Folch PSUP and centrifuged. The upper phase was removed and the lower phase was evaporated under a stream of nitrogen, re-suspended in 5 ml of CHCl_3_:CH_3_OH (2:1 by vol), and stored at 4°C.

### Qualitative and quantitative analysis of plasma lipids

Neutral and acidic lipids were analyzed qualitatively by high-performance thin-layer chromatography (HPTLC) following modifications of previously described methods [7,51,52,56]. Lipids were spotted on 10 × 20 Silica gel 60 HPTLC plates (E. Merck, Darmstadt, Germany) using a Camag Linomat III auto-TLC spotter (Camag Scientific Inc., Wilmington, NC, USA). The amount of plasma per lane was equivalent to 15 μl for acidic lipids and 2.5 μl for neutral lipids. To enhance precision, an internal standard (oleoyl alcohol) was added to the neutral and acidic lipid standards and the plasma samples as previously described [52]. Purified lipid standards were purchased from Matreya, Inc. (Pleasant Gap, PA, USA), Avanti Polar Lipids, Inc. (Alabaster, AL, USA), and Sigma (St. Louis, MO, USA).

For neutral and acidic lipids, the plate was developed to a height of 4.5 and 6.0 cm, respectively, with chloroform : methanol : acetic acid : formic acid : water (35:15:6:2:1 by vol), and was then developed to the top with hexanes : diisopropyl ether : acetic acid (65:35:2 by vol). Neutral and acidic lipids were visualized by charring with 3% cupric acetate in 8% phosphoric acid solution, followed by heating in an oven at 160–170°C for 7 min as previously described [7,51,52,56].

The density and percentage distribution of the individual lipid bands was determined by scanning the plate on a Personal Densitometer SI with ImageQuant software (Molecular Dynamics, Sunnyvale, CA, USA) for neutral and acidic lipids. The density values for each neutral and acidic lipid were fit to a standard curve of the respective lipid and used to calculate individual concentrations as described previously [52]. All plasma lipid concentrations are expressed as milligram of lipid per milliliter of plasma.

### Statistical analysis

Analysis of variance (ANOVA) followed by a Fisher's protected least significant difference (PLSD) test were used to evaluate the significance of differences between the UR, R, and R-RF groups. A paired *t*-test was used to analyze differences within the R-RF group (Statview, v. 5.0) [57]. In each figure, *n *designates the number of individual mice analyzed.

## Abbreviations

Adenosine triphosphate (ATP)

Caloric restriction (CR)

High-density lipoprotein (HDL)

High-performance thin-layer chromatography (HPTLC)

Low-density lipoprotein (LDL)

Restricted (R)

Restricted/Re-fed (R-RF)

Unrestricted (UR)

## Competing interests

The author(s) declare that they have no competing interests.

## Authors' contributions

LBM participated in the design of the study, carried out the study, performed the lipid analysis, performed the statistical analysis, and helped to draft the manuscript. CAD participated in the design of the study, performed statistical analysis, and coordinated and helped to draft the manuscript. TNS designed the study and helped to draft the manuscript. All authors read and approved the final manuscript.
